# Contribution of Capsule Endoscopy Early in a Bleeding Episode to Treatment of Small Bowel Angioectasia: A Case Report

**DOI:** 10.3390/medicina57040321

**Published:** 2021-03-31

**Authors:** Yoshinori Arai, Maiko Ogawa, Rikako Arimoto, Yoshitaka Ando, Daisuke Endo, Tatsuya Nakada, Ichiro Sugawara, Hiroshi Yokoyama, Keiko Shimoyama, Hiroko Inomata, Yosuke Kawahara, Masayuki Kato, Seiji Arihiro, Atsushi Hokari, Masayuki Saruta

**Affiliations:** 1 Division of Gastroenterology and Hepatology, Department of Internal Medicine, The Jikei University Katsushika Medical Center, Tokyo 105-8461, Japan; ms04ogawa@jikei.ac.jp (M.O.); rikakoyabuki@gmail.com (R.A.); m06a007f@yahoo.co.jp (Y.A.); enddai1209@yahoo.co.jp (D.E.); nakatatsu55@hotmail.com (T.N.); qq6z4sed@yahoo.co.jp (I.S.); dozeen1@gmail.com (H.Y.); sarihiro@gmail.com (S.A.); hokari_a@nifty.com (A.H.); 2 Tekko Building Marunouchi Clinic, Tokyo 100-0005, Japan; 3 Department of Endoscopy, The Jikei University Katsushika Medical Center, Tokyo 105-8461, Japan; keikos_29@yahoo.co.jp (K.S.); hirokomoko@hotmail.co.jp (H.I.); divemusashi@gmail.com (Y.K.); masakato89@gmail.com (M.K.); 4 Division of Gastroenterology and Hepatology, Department of Internal Medicine, The Jikei University School of Medicine, Tokyo 105-8461, Japan; m.saruta@jikei.ac.jp

**Keywords:** small bowel capsule endoscopy, balloon-assisted endoscopy, small bowel angioectasia, small bowel bleeding, argon plasma coagulation

## Abstract

*Background*: Recent advances in endoscopic devices such as small bowel capsule endoscopy and balloon-assisted endoscopy have improved the level of medical care for small bowel bleeding. However, treating small bowel angioectasia remains challenging because repeated intermittent bleeding can occur from the multiple minute lesions (about 1 mm in size) that develop in a synchronous and metachronous manner. Here, we report a case of small bowel angioectasia in which capsule endoscopy performed early in a bleeding episode contributed to treatment. *Case Summary*: A 66-year-old man with suspected small bowel bleeding underwent small bowel capsule endoscopy and balloon-assisted endoscopy with argon plasma coagulation hemostasis for a small intestinal angioectasia. Because small bowel bleeding recurred intermittently after the treatment, small bowel capsule endoscopy and balloon-assisted endoscopy were repeated when there was no bleeding, but no abnormalities were found. Subsequent small bowel capsule endoscopy during a bleeding episode revealed bloody intestinal fluid in the proximal small intestine. Peroral balloon-assisted endoscopy was performed 2 days after SBCE for detailed observation of the small intestinal mucosa at the suspected bleeding site, and there a 1-mm Dieulafoy’s lesion with no active bleeding was identified. We performed argon plasma coagulation, and no bleeding was observed thereafter. *Conclusions*: Small bowel capsule endoscopy immediately after bleeding onset can identify the bleeding source of multiple minute lesions in small bowel angioectasia.

## 1. Introduction

Gastrointestinal bleeding is reported to occur in 50–150 per 100,000 population, and is often encountered in daily clinical practice [1]. Medical care for gastrointestinal bleeding consists of identification of the bleeding site and diagnostic evaluation and treatment of hemorrhagic lesions. Recent advances in endoscopic devices have improved the level of medical care for gastrointestinal bleeding. Observation of the entire gastrointestinal tract and diagnostic evaluation and treatment of hemorrhagic lesions has become possible with the full use of upper gastrointestinal endoscopy, colonoscopy, small bowel capsule endoscopy (SBCE), and balloon-assisted endoscopy (BAE).

Treating small bowel angioectasia, however, can still be challenging in daily clinical practice because repeated intermittent bleeding can occur from the multiple minute lesions (about 1 mm in size) that develop in a synchronous and metachronous manner. The rebleeding rate after angioectasia treatment remains high, at 28.0–33.8% [2,3,4]. One reason for this is the difficulty identifying such minute lesions by SBCE and BAE. The other reason for this is the difficulty of identifying which of the multiple angioectatic lesions is the source of bleeding, because spontaneous hemostasis sometimes occurs during examination. Moreover, since the small intestinal mucosa regenerates fastest of any tissue in the body, the hemorrhagic lesion is covered by regenerated epithelium within 3–5 days and becomes difficult to identify [5].

Given that it is essential to accurately identify the bleeding source from among the multiple minute lesions in small bowel angioectasia, SBCE should be performed immediately after bleeding onset [6,7]. Detection of blood or bloody intestinal fluid by SBCE is an important finding indicating the bleeding site; then, BAE should be performed for detailed observation of the small intestinal mucosa at the suspected bleeding site, enabling the bleeding source to be identified and treated from among multiple minute lesions in small bowel angioectasia.

## 2. Case Presentation

The patient was a 66-year-old man with a chief complaint of the passage of tarry stools. His past medical history included atrial fibrillation and arteriosclerosis obliterans, for which he was taking antithrombotic agents (clopidogrel sulfate and aspirin enteric-coated tablets). He was on hemodialysis for diabetic nephropathy.

**Figure 1:**
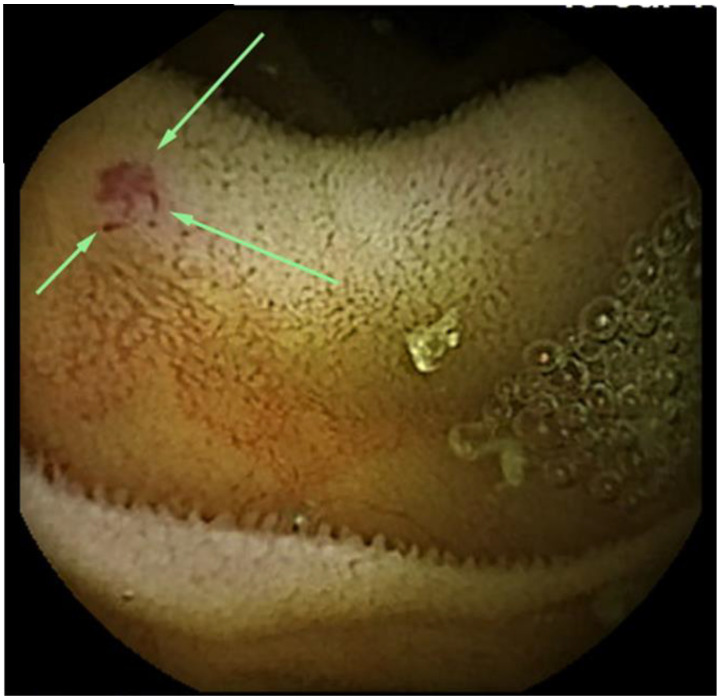
Small bowel capsule endoscopy findings at first examination. A 2-mm angioectasia (arrows) is apparent in the proximal small intestine, with no bleeding source identified.

**Figure 2:**
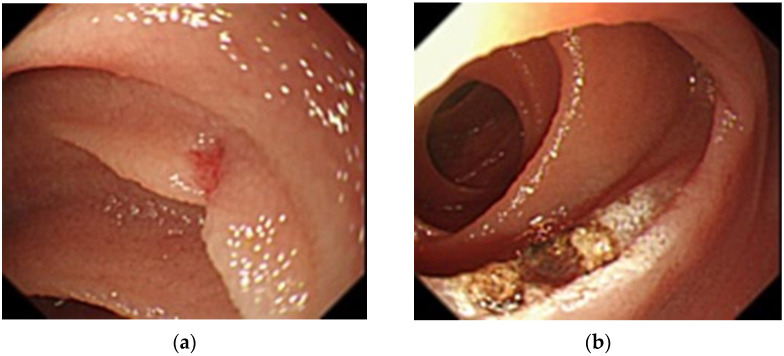
Balloon-assisted endoscopy findings at first examination. (**a**): A 2-mm angioectasia 120 cm distal to the ligament of Treitz in the small intestine with no bleeding source identified; (**b**): Argon plasma coagulation for the angioectasia.

After confirming anemia (hemoglobin [Hb] level 6.8 g/dL), we diagnosed gastrointestinal bleeding. No abnormalities were found on upper gastrointestinal endoscopy and colonoscopy. We suspected small bowel bleeding (SBB) and performed SBCE (PillCamTM SB3 Capsule, Medtronic, Minneapolis, MN, USA) 7 days after the last tarry stool. We identified 2-mm angioectasia (Yano-Yamamoto classification Type 1b [8]) in the proximal small intestine during SBCE, and no blood retention was seen within the intestinal lumen (Figure 1). Peroral BAE was performed, and argon plasma coagulation (APC) was performed for this lesion (Figure 2).

However, the patient intermittently passed tarry stool after the treatment (about once every 2 weeks) and blood transfusion was required every time for anemia (Hb 6–7 g/dL). Enteric-coated aspirin was discontinued while antithrombotic therapy with clopidogrel sulfate was continued, but bleeding episodes occurred repeatedly. Peroral BAE and SBCE were performed at 4 months and 6 months after the treatment, respectively, with no abnormalities identified during either procedure. We suspected the reason for not detecting abnormal findings was probably that these endoscopies were performed when there was no bleeding (at least 7 days after the last tarry stool). Therefore, we asked the patient to visit us immediately after bleeding onset.

**Figure 3:**
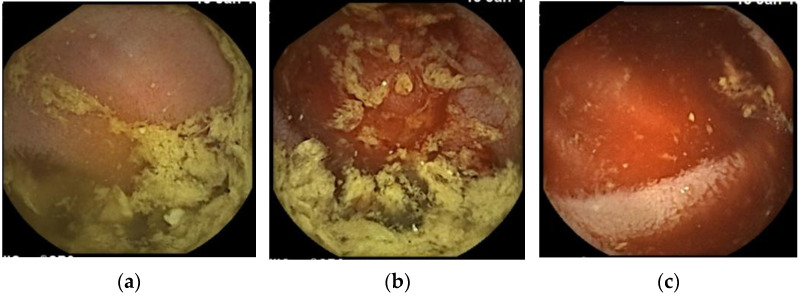
Small bowel capsule endoscopy findings at third examination. (**a**): Yellow and transparent intestinal fluid; (**b**): Boundary where the color of the intestinal fluid changes. Intestinal fluid on the proximal side to the boundary is yellow and transparent, while that on the distal side is bloody; (**c**): Bloody intestinal fluid.

**Figure 4:**
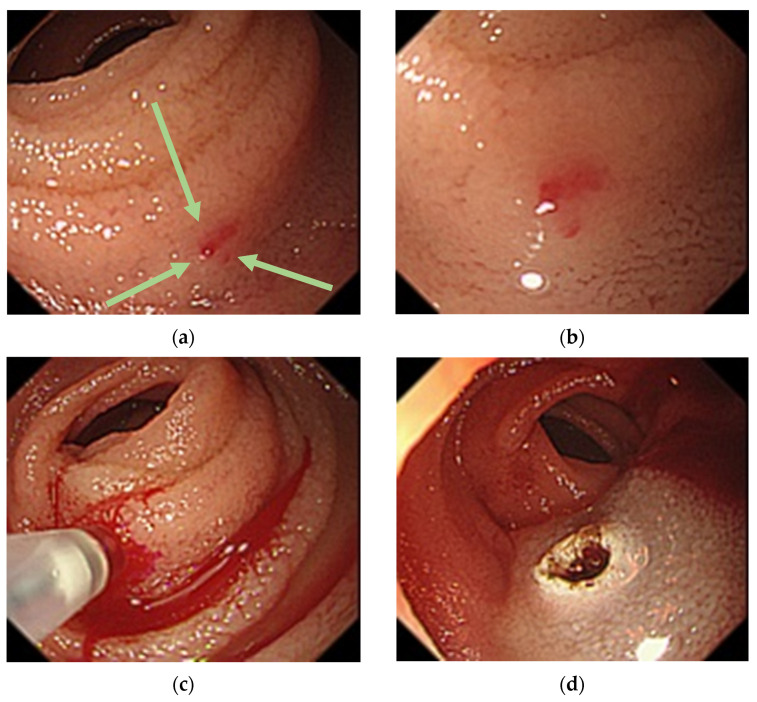
Balloon-assisted endoscopy findings at third examination. (**a**): A 1-mm Dieulafoy’s lesion (arrows) 150 cm distal to the ligament of Treitz in the small intestine; (**b**): Near-field image showing the lesion; (**c**): Overt bleeding caused by local injection of physiological saline; (**d**): Argon plasma coagulation for the lesion.

The patient passed a large amount of tarry stool 7 months after the treatment and visited us immediately. He was urgently admitted because of anemia (Hb 6.9 g/dL; Table 1). We performed SBCE 13 h after he had passed the tarry stool and found bloody intestinal fluid in the proximal intestine (Figure 3). The intestinal fluid in the lumen proximal to this site was yellow and transparent, while that in the lumen distal to this site was bloody. A qualitative diagnosis of hemorrhagic lesion was difficult due to the presence of the bloody intestinal fluid and residue, but a hemorrhagic lesion was likely present at this site. Peroral BAE was performed 2 days after SBCE for detailed observation of the small intestinal mucosa at the suspected bleeding site, and there we identified a 1-mm Dieulafoy’s lesion, which was 150 cm distal to the ligament of Treitz (Figure 4). We injected physiological saline near the lesion to prevent perforation, then performed APC for the lesion. No rebleeding has occurred 18 months after the second APC treatment.

We obtained written informed consent from the patient for inclusion in this report. Ethical review and approval were waived for this study because our institution does not require these for case reports.

Abbreviations: ALP, alkaline phosphatase; AST, aspartate aminotransferase; BUN, blood urea nitrogen; CK, creatine kinase; CRP, C-reactive protein; LDH, lactate dehydrogenase; MCV, mean corpuscular volume; RBC, red blood cell count; TP, total protein; WBC, white blood cell count; Hb, hemoglobin; Ht, hematocrit; Plt, platelet; ALT, alanine aminotransferase; γ-GTP, γ-glutamyl transpeptidase; Alb, albumin; Cr, creatinine; Cl, chloride.

## 3. Discussion

Medical care for SBB has improved since observation of the entire small intestine became possible with the introduction of SBCE and BAE [9,10,11,12]. However, we still often encounter SBB cases that are difficult to treat. These difficult cases are often characterized by minute hemorrhagic lesions (approximately 1 mm in size) and intermittent bleeding (repeated cycles of bleeding and hemostasis).

Regarding the first characteristic, because SBCE captures 2–6 images per second, minute lesions of about 1 mm in size may be missed or may be hidden by air bubbles or bile in the small intestinal lumen [13,14]. Moreover, it is difficult to observe the entire small intestinal mucosa in detail and to detect minute lesions by BAE due to peristaltic movement of the small intestine and technical difficulties in BAE.

In terms of the second characteristic, it is hard to detect the bleeding site by SBCE in the absence of active bleeding because the bleeding site is identified based on the presence of bloody fluid. SBCE, therefore, should be performed during active bleeding. Blood in the intestinal tract flows toward the anal side; thus, the bleeding source should be at the site where bloody fluid is first observed during SBCE. However, qualitative diagnosis of the bleeding source using SBCE is often difficult because minute lesions can be covered by bloody intestinal fluid. Nakamura et al. reported that the rate of successful qualitative diagnosis of the bleeding source based on SBCE findings alone was low (11.2%) [15]. In our opinion, the disadvantages of BAE as a diagnostic modality include the following: (1) endoscopic insufflation during examination increases the pressure in the intestinal tract, which then stops the bleeding; and (2) endoscopic insufflation moves bloody fluid from the bleeding site, obscuring the bleeding point. SBCE, on the other hand, does not require air insufflation and thus does not affect the pressure in the intestinal tract, so the small intestine can be observed in the natural state and the bleeding site can be identified using bloody intestinal fluid as an index. Therefore, performing SBCE immediately after bleeding onset is crucial for identifying the bleeding site [16,17]. Regarding the timing of performing SBCE in OGIB, Esaki et al. reported that SBCE within 7 days after the last bleeding was an independent factor associated with the diagnostic yield of SBCE [18]. In their study, various findings, such as inflammatory and neoplastic lesions were included in the analysis, and the size of hemorrhagic lesions was not investigated. Moreover, non-specific mucosal changes including minute red spots were not regarded as findings in the study. After spontaneous hemostasis, the hemorrhagic lesion is covered by the regenerated epithelium within 3–5 days [5], meaning that minute hemorrhagic lesions like the one in the present case may not be detected if SBCE is performed a few days after the last bleeding. Therefore, SBCE should be performed during active bleeding before the regenerated epithelium of the intestine makes the source of bleeding undetectable. If the bleeding site is identified by SBCE, the suspected bleeding site should be evaluated carefully by BAE, and minute hemorrhagic lesions can then be diagnosed and treated accordingly.

Hemorrhagic lesions of the small intestine are divided into three categories: vascular, inflammatory, and neoplastic lesions. Angioectasia is of the vascular type, with dilated capillaries in the lamina propria and submucosa, and it accounts for 23–52% of hemorrhagic lesions [3,9,19,20,21]. Small bowel angioectasia is an acquired disease often seen in patients with valvular heart disease, diabetes, or renal disease [22,23]. These patients often receive antithrombotic therapy for underlying conditions, which leads to overt bleeding requiring treatment in some cases, although not a few patients are asymptomatic without bleeding [21]. Multiple synchronous and metachronous lesions often occur, and even after treatment at one site, bleeding may occur at other sites [2,4,24,25]. The rebleeding rate after initial treatment for small bowel vascular lesions is high (28.0–33.8%) [2,3,4]. In addition, Sakai et al. reported that 83.8% of patients with angioectasia have multiple lesions, with ≥3 angioectatic lesions posing a high risk of rebleeding [3].

In this case, the patient was on hemodialysis for diabetic nephropathy and his past medical history included atrial fibrillation and arteriosclerosis obliterans, for which he was taking antithrombotic agents; therefore, he had various risk factors for small bowel angioectasia and bleeding. Angioectasias were seen at two different sites over time. Treatment was not effective initially, but a second treatment was. At the first treatment, SBCE was performed during inactive bleeding and only one angioectatic lesion was identified, which was not the main bleeding source. At the second treatment, SBCE was performed during active bleeding, and the minute hemorrhagic lesion was successfully identified and treated by BAE. Both SBCE and BAE were performed three times in the present case, and the finding of SBCE performed during active bleeding, not inactive bleeding, contributed to treatment. It is difficult to identify which of the multiple angioectatic lesions are the source of bleeding. SBCE during active bleeding is useful in identifying the bleeding site, then BAE should be performed for detailed observation of the small intestinal mucosa at the suspected bleeding site, thus enabling the bleeding source to be identified and treated from among the multiple minute lesions in small bowel angioectasia. SBCE should be performed immediately if rebleeding is observed. Repeating this sequence of examinations and treatments is likely to decrease the rebleeding rate in small bowel angioectasia. To achieve this, it is important to advise patients with small bowel angioectasia to immediately see a doctor if bleeding recurs and to establish a system that enables SBCE to be performed immediately when the patient visits with rebleeding.

## 4. Conclusions

Treating small bowel angioectasia remains challenging because repeated intermittent bleeding can occur from the multiple minute lesions (about 1 mm in size) that develop in a synchronous and metachronous manner. Small bowel capsule endoscopy immediately after bleeding onset can identify the source of bleeding in small bowel angioectasia.

## Figures and Tables

**Table 1:** Blood test findings on admission.

Blood Count		Blood Biochemistry			
WBC (/μL)	5100	AST (IU/L)	8	Na (mEq/L)	142
RBC (/μL)	232 × 10^4^	ALT (IU/L)	7	K (mEq/L)	5.0
Hb (g/dL)	6.9	LDH (IU/L)	114	Cl (mEq/L)	103
MCV (fL)	93.1	T-Bil (mg/dL)	0.3	CRP (mg/dL)	0.46
Ht	21.6%	ALP (IU/L)	234		
Plt (/μL)	19.5 × 10^4^	γ-GTP (IU/L)	32		
		TP (g/dL)	5.3		
		Alb (g/dL)	2.9		
		CK (IU/L)	12		
		BUN (mg/dL)	56		
		Cr (mg/dL)	6.26		

Abbreviations: ALP, alkaline phosphatase; AST, aspartate aminotransferase; BUN, blood urea ni-trogen; CK, creatine kinase; CRP, C-reactive protein; LDH, lactate dehydrogenase; MCV, mean corpuscular volume; RBC, red blood cell count; TP, total protein; WBC, white blood cell count; Hb, hemoglobin; Ht, hematocrit; Plt, platelet; ALT, alanine aminotransferase; γ-GTP, γ-glutamyl transpeptidase; Alb, albumin; Cr, creatinine; Cl, chloride.

## Data Availability

No new data were created or analyzed in this study. Data sharing is not applicable to this article.
